# Relationship between CCL25/CCR9 Levels in Follicular Fluid and High Ovarian Response in Patients with Polycystic Ovary Syndrome

**DOI:** 10.1155/2024/2449037

**Published:** 2024-10-03

**Authors:** Yongxin Hao, Qianqian Yin, Fangfang Hu, Xiaoyan Liu, Yaru Yang, Fang Sun, Xiaonan Yan

**Affiliations:** ^1^Affiliated Xuzhou Clinical College of Xuzhou Medical University, Xuzhou, China; ^2^Clinical Center of Reproductive Medicine, Xuzhou Central Hospital, Xuzhou 221000, China; ^3^Department of Obstetrics and Gynecology, Xuzhou Central Hospital, Xuzhou 221000, China

## Abstract

**Objective:**

Polycystic ovary syndrome (PCOS) is one of the prevalent gynecological endocrine disorders encountered in clinical practice. Women diagnosed with PCOS demonstrate increased ovarian responsiveness, rendering them more prone to ovarian hyperstimulation syndrome (OHSS) during controlled ovarian stimulation (COS) procedures. The current study aimed at investigating whether CCL25/CCR9 plays a role in the pathological process of high ovarian response (HOR) during COS procedures.

**Design:**

Single-center retrospective cohort study. *Patients*. A total of 200 PCOS patients who received a fixed regimen of gonadotropin-releasing hormone (GnRH) antagonist were enrolled in this study. The cohort comprised 118 patients exhibiting HOR and 82 patients demonstrating a normal ovarian response (NOR).

**Results:**

The age and body mass index (BMI) variances across the two groups did not differ significantly. Similarly, the two groups observed no statistically significant differences in the baseline levels of luteinizing hormone (LH), progesterone (P), estradiol (E_2_), basal prolactin (PRL), and testosterone (T). Compared to the NOR group, HOR patients exhibit markedly elevated levels of anti-Müllerian hormone (AMH), antral follicle count (AFC), basal follicle-stimulating hormone (FSH), and HOMA-IR (all *p*  <  0.05). Conversely, no statistically significant differences were observed between the two groups with respect to COS parameters, encompassing initial gonadotropin (Gn) dose, stimulation duration, and total Gn dose. During COS, the number of oocytes with diameter ≥14 mm, the levels of E_2_ on the HCG day, and the number of retrieved oocytes were significantly higher in the HOR group than in the NOR group (all *p* < 0.001). Additionally, the levels of CCL25/CCR9, matrix metalloproteinases (MMPs), tissue inhibitor of metalloproteinases (TIMPs), TNF-*α*, and IL-6 were significantly higher in the FF of the HOR group than in the NOR group (all *p* < 0. 001), while the variance in IL-1*β* levels between the two cohorts did not reach statistical significance. The relevance analysis results indicated that the levels of CCL25/CCR9 in the FF of the HOR group are positively correlated with the number of retrieved oocytes and oocytes with diameters ≥14 mm during COS, AMH levels, and AFC. Concurrently, the CCL25 levels in the FF of the HOR group were positively correlated with HOMA-IR. Multivariable linear regression analysis revealed that the elevated AFC and HOMA-IR independently increase the CCL25 levels.

**Conclusion:**

The CCL25/CCR9 levels in FF are positively correlated with the clinical indicators of HOR, suggesting that CCL25/CCR9 may play a role in the pathogenesis of HOR in patients with PCOS.

## 1. Introduction

PCOS is one of the most prevalent gynecological endocrine disorders among women of reproductive age and is characterized by menstrual irregularities, hyperandrogenism, and polycystic ovarian morphology [1]. PCOS is considered a heterogeneous condition, influenced by various genetic factors, endocrine functions, and the environment; however, the underlying mechanisms are yet to be elucidated [2]. PCOS patients exhibit heightened sensitivity during ovulation induction treatments, as the ovaries may respond uncontrollably to gonadotropins, increasing the risk of ovarian hyperstimulation syndrome (OHSS). The incidence of OHSS reaches up to 45% during ovulation induction in PCOS patients [3], resulting in assisted reproductive failure or adverse effects on maternal health [4]. Some studies suggested that the intrinsic cause of this failure may be the presence of multiple small follicles in the ovaries of PCOS patients who do not undergo development [5]. Controlled ovarian stimulation (COS) is highly prone to the simultaneous development of several follicles. In recent years, PCOS has exhibited a trend of younger age of onset, rendering them more likely to respond to ovulation-inducing medications [6]. Chen et al. showed that HCG stimulates the synthesis of the vascular endothelial growth factor (VEGF) and cytokines in the ovaries, activating the inflammatory pathways, leading to a high ovarian response (HOR), and ultimately causing OHSS [7]. However, the specific mechanisms underlying this phenomenon are yet to be clarified.

In 1980, Espey et al. hypothesized that ovulation represents an inflammatory reaction [8]. In a physiological state, various inflammatory mediators, such as the interleukin (IL)-1 family, IL-6, tumor necrosis factor-alpha (TNF-*α*), granulocyte-macrophage colony-stimulating factor (GM-CSF), and chemokines, play a significant role in modulating follicular development across various stages during the ovulation process [9]. These inflammatory mediators regulate hormone secretion along the hypothalamic-pituitary-ovarian (HPO) axis, thereby promoting follicular development, maturation, and ovulation [10]. Several studies have shown elevated levels of cytokines in the peripheral circulation and follicular fluid (FF) of patients with PCOS [11, 12]. Specifically, obesity coupled with higher levels of androgens or insulin resistance (IR) may induce persistent immune system stimulation in PCOS patients, leading to chronic low-grade inflammation, which has an impact on follicular development and ovulation, ultimately resulting in infertility. Inflammatory factors also play an important role in the pathogenesis of OHSS. The pathophysiological mechanism of OHSS involves impaired systemic vascular endothelial function induced by an inflammatory reaction, leading to microvascular leakage [13].

C-C chemokine receptor 9 (CCR9) is expressed on the cell membrane of several immune cells, including dendritic cells, neutrophils, lymphocytes, monocytes, and vascular endothelial cells. On the other hand, C-C chemokine ligand 25 (CCL25) is predominantly expressed in the thymic epithelium and can also be produced by other parenchymal cells [14]. CCR9 is the sole identified receptor for CCL25, and both molecules engage in interactions that play pivotal roles in various pathological and physiological processes within the body [15]. Another study on ovarian cancer showed a significant increase in CCR9 expression in seromucous carcinoma tissues compared to nontumorous tissues. Further in vitro cell studies demonstrated a crucial role of CCL25-CCR9 interaction in the invasion and migration of ovarian cancer cells [16]. Duffy et al. revealed that ovarian follicular membrane cells attracted monocytes, B cells, T cells, and natural killer cells to the local ovarian environment through CCL25 expression and that these cells are actively participating in the normal ovulation process. These studies underscore the pivotal roles of the chemotactic factors in ovarian pathophysiology [17]. Nevertheless, whether the CCL25/CCR9 axis is involved in the pathogenic mechanisms of the HOR in PCOS patients undergoing COS is yet unexplored.

In previous experiments, we observed the upregulation levels of CCL25 and CCR9 in FF among individuals diagnosed with PCOS compared to the control group. Therefore, this present study aimed at exploring the relationship between the levels of CCL25/CCR9 in FF and HOR in PCOS patients.

## 2. Materials and Methods

### 2.1. Participants

This retrospective study included infertile patients with polycystic ovary syndrome who underwent in vitro fertilization (IVF) between November 2021 and October 2023 at the Reproductive Medicine Centre of Xuzhou Central Hospital, Jiangsu, China. It was approved by the Ethical Review Committee of Xuzhou Central Hospital (Approval No. XZXY-LK-20210915-054), and informed consent was obtained from each participant before they participated in the study.

A cohort of 200 Chinese Han women, aged 25–40 years and diagnosed with PCOS based on the Rotterdam Criteria, was assembled from Xuzhou Central Hospital for this study [18]. The exclusion criteria were as follows: (1) the presence of other disorders causing elevated androgens, anovulation, or polycystic ovarian changes; (2) history of ovarian tumors, endometriomas, endometriosis, ovarian surgery, or radiotherapy and chemotherapy; (3) history of autoimmune diseases; (4) patients experiencing thyroid and adrenal disorders and hyperprolactinemia concomitantly; (5) intake of any medications affecting reproductive hormone levels, blood glucose, and lipid profiles in the three months prior to participation in the study.

Based on the defined criteria, PCOS patients were categorized into the HOR group and normal ovarian response (NOR) groups. The HOR is defined as patients exhibiting at least one of the following features [19]: (1) acquisition of >15 oocytes during a COS cycle or the termination of the cycle due to excessive follicular growth; (2) retrieval of >20 oocytes with a diameter of ≥14 mm in a COS cycle; or (3) manifestation of moderate to severe OHSS following COS. Conversely, PCOS patients with a poor ovarian response (POR) were excluded from the study. The POR was characterized if a minimum of two out of the three specified characteristics were detected [20]: (1) maternal age ≥40 years or any other identified risk factor for POR; (2) prior history of POR; or (3) anomalous results on ovarian reserve testing. The remaining cases were categorized as the NOR.

### 2.2. COS Protocols

This study exclusively enrolled patients undergoing a standardized gonadotropin-releasing hormone (GnRH) antagonist protocol. Recombinant human Gn (Gonal-F, Merck Serono, Germany) was initiated on menstrual days 2–3, and the starting dosage was determined based on the patient age, baseline follicle-stimulating hormone (FSH) levels, and antral follicle count (AFC). The regular monitoring of follicle diameters, endometrial thickness, and serum hormone levels facilitated timely adjustments to the Gn dosage. When the dominant follicle achieved a diameter of ≥14 mm, 0.25 mg/day GnRH antagonist (Cetrotide, Hybio, China) was added until the trigger day, ceasing Gn administration when at least two follicles reached ≥18 mm or the average diameter of three follicles reached ≥17 mm. 6000–10000 U HCG (Livzon, China) was injected intramuscularly, and oocytes were retrieved 34–36 h postinjection. Data regarding the initial and total Gn dose, the stimulation duration, the number of oocytes with diameter ≥14 mm during COS, the estradiol (E_2_) levels, and the number of oocytes were retrieved.

### 2.3. Hormone Estimation and Pelvic Ultrasonography

Peripheral blood samples were collected from the study participants during 1–5 menstrual cycle days to assess the following serum parameters: anti-Müllerian hormone (AMH), basal FSH, basal luteinizing hormone, basal progesterone, basal estradiol, basal prolactin, and basal testosterone (T). Blood samples were withdrawn from the elbow vein on the day of HCG to assess the levels of E_2_. Ultrasound examination was used to assess both the AFC and the morphology of polycystic ovaries and to monitor follicle development during COS.

### 2.4. Sample Collection and Measurements

Blood-free FF was obtained from dominant follicles harboring oocytes (approximately 18–20 mm in diameter) during an in vitro fertilization/intracytoplasmic monosperm microinjection and embryo transfer (IVF/ICSI-ET) cycle, according to the oocyte extraction protocol. Subsequently, the FFs were clarified by centrifugation at 600 rpm for 10 min at room temperature and preserved at −80°C until further analysis [21]. The general information of all the participants, including age, height, weight, and body mass index, was collected.

### 2.5. Enzyme-Linked Immunosorbent Assay (ELISA)

The levels of CCL25, CCR9, matrix metalloproteinase (MMP)-2, MMP-9, tissue inhibitor of metalloproteinase (TIMP)-1, TIMP-2, IL-6, IL-1*β*, and TNF-*α* were quantified using ELISA kits obtained from EIAab (Wuhan, China), according to the manufacturer's instructions. A volume of 100 *μ*L FF was incubated with specific antibodies to detect the target molecules. Finally, the immunodetection of the samples was conducted at 450 nm optical density.

### 2.6. Statistical Analysis

Statistical analysis was carried out using SPSS software, version 29.0.1. Data are presented as mean ± standard deviation or median (interquartile range) as appropriate. The normal distribution of continuous variables was assessed using the Kolmogorov–Smirnov test. Student's *t*-test was employed for two normally distributed independent samples, while the Mann–Whitney *U*-test was applied for non-normally distributed variables. The correlations were examined through Spearman's rank correlation analysis. Multifactorial linear regression analysis was conducted to identify the factors associated with elevated CCL25 levels in PCOS patients exhibiting a HOR. All analyses were two-sided, and *p* < 0.05 was considered statistically significant.

## 3. Results

### 3.1. Clinical Characteristics and Reproductive Situation between Different Ovarian Response Groups in PCOS

The present study comprised 200 women diagnosed with PCOS, all of whom underwent COS using a fixed protocol of GnRH antagonist.  Table 1 summarizes the clinical characteristics of the subjects. Interestingly, the AMH (*p* < 0.001), AFC (*p* < 0.001), basal FSH (*p*=0.03), and homeostasis model assessment-insulin resistance (HOMA-IR) (*p*=0.039) were significantly higher in the HOR group than in the NOR group, whereas the other baseline characteristics did not differ significantly between the two groups (all *p* > 0.05).

A comparison between the reproductive parameters indicated no statistically significant disparities in the initial dosage of Gn, duration of stimulation, or total Gn dosage administered in the two groups (all *p* > 0.05). Notably, the number of oocytes with a diameter of ≥14 mm during COS, the E_2_ level on the HCG day, and the number of oocytes retrieved were significantly higher in the HOR group than in the NOR group (all *p* < 0.001). The details of the reproductive situation in the two groups are summarized in Table 1.

### 3.2. Levels of CCL25/CCR9, MMPs and Their Inhibitors, and Cytokines in FF of PCOS Patients in Different Ovarian Response Groups

**Figure 1:**
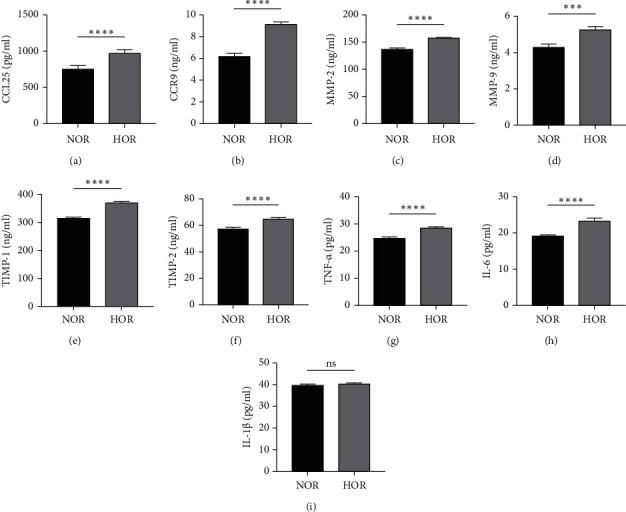
Levels of CCL25/CCR9, MMPs and their inhibitors, and cytokines in the FF of the HOR and NOR groups. (a) CCL25 levels; (b) CCR9 levels; (c) MMP-2 levels; (d) MMP-9 levels; (e) TIMP-1 levels; (f) TIMP-2 levels; (g) TNF-*α* levels; (h) IL-6 levels; (i) IL-1*β* levels. The data are shown as the means ± SEM. ^∗∗∗^*P* < 0.001, ^∗∗∗∗^*P* < 0.0001, ns >0.05.

The levels of CCL25/CCR9, MMPs, TIMPs, and the related cytokines IL-6, IL-1*β*, and TNF-*α* were detected in the FF of both groups. Figures 1(a) and 1(b) show the significantly elevated levels of CCL25 and CCR9 in the FF of the HOR group than in the NOR group (all *p* < 0.001). Additionally, the levels of MMP-2 and MMP-9 were significantly elevated in the HOR group compared to the NOR group (all *p* < 0.001). Similar results were observed in the case of TIMP-2 and TIMP-1 inhibitors (all *p* < 0.001) (Figures 1(c), 1(d), 1(e), and 1(f)). Furthermore, the concentrations of TNF-*α* and IL-6 increased significantly (all *p* < 0.001) in women in the HOR group compared to those in the NOR group (Figures 1(g) and 1(h)), while the IL-1*β* level did not vary significantly between the two groups (Figure 1(i)).

### 3.3. Correlation between Clinical Indicators of Ovarian Response and CCL25/CCR9 and MMPs in FF

In order to investigate the potential involvement of CCL25/CCR9 and MMPs in the pathogenesis of HOR among patients diagnosed with PCOS, the correlations between CCL25/CCR9, MMPs and AMH, AFC, the number of oocytes with a diameter of ≥14 mm during COS, E_2_ on the HCG day, the number of oocytes retrieved, and HOMA-IR were investigated (Table 2) using Spearman's rank correlation analyses. In the HOR group, CCL25 and CCR9 were positively correlated with the number of oocytes retrieved (*p*=0.042 and *p*=0.02, respectively), the number of oocytes with diameter ≥14 mm during COS (*p*=0.038 and *p*=0.003, respectively), AMH (*p* < 0.001 and *p*=0.004, respectively), and AFC (all *p* < 0.001). Furthermore, a positive correlation was established between CCL25 and HOMA-IR (*p* < 0.001). MMP-2 and MMP-9 expression levels were positively correlated with the number of oocytes retrieved (*p*=0.003 and *p*=0.048, respectively), the number of oocytes with diameter ≥14 mm during COS (*p*=0.009 and *p* < 0.001, respectively), and AMH (all *p* < 0.001). A positive correlation was also observed between MMP-9 and AFC (*p*=0.002).

A linear regression analysis using CCL25 level as the dependent variable and AMH, AFC, and HOMA-IR as independent variables (Table 3) revealed that the elevated AFC and HOMA-IR levels were independently correlated with increasing CCL25 levels in HOR patients (*p*=0.010 and *p* < 0.001, respectively).

## 4. Discussion

PCOS is characterized by ovulation disorders and is prone to increased ovarian response and OHSS during COS [22]. Previous studies have shown that HCG stimulated the inflammatory factors in the ovaries and activated inflammatory pathways, leading to HOR and OHSS [23, 24]. Some studies indicated that CCR9 is involved in physiological processes, including the migration and maturation of inflammatory cells, and that the CCL25/CCR9 axis plays a significant role in both inflammatory and noninflammatory diseases [15]. This present study aimed at investigating the relationship between the levels of CCL25/CCR9 in FF and HOR in PCOS patients. The results indicated the higher levels of CCL25 and CCR9 in the FF of PCOS women with HOR than those of PCOS women with NOR during COS; also, CCL25/CCR9 was positively correlated with the clinical indicators for the diagnosing of HOR.

In comparison with patients with HOR, those with NOR experienced a significantly reduced cancellation rate for fresh embryo transfer (ET) and a lower incidence of severe OHSS. Researchers sought to identify the clinical indicators predicting the HOR to enhance the identification and management of HOR patients and reduce the occurrence of OHSS [25]. Another study deemed AMH and AFC served as the indicators for ovarian hyperstimulation during COH [26]. Accumulating evidence suggested that serum inhibit-B can predict the HOR before COS, while the prediction can be based on the serum E_2_ concentration and the number of developing follicles during COS [27]. Herein, we compared the characteristics between the two groups of patients and found that AMH and AFC were significantly higher than those in the NOR group. The comparison of the COS situations between the two groups of patients revealed that the levels of E_2_ on the HCG day, the number of follicles with diameter ≥14 mm during COS, and the number of oocytes retrieved were significantly higher in the HOR group than in the NOR group.

Chemokines and their receptors orchestrate the localization and mobilization of immune cells, collectively contributing to various pathological and physiological processes in the body. A review described the involvement of CCL25/CCR9 in various pathological processes, such as inflammatory diseases, autoimmune diseases, metabolic disorders, and tumorigenesis [15]. For example, the severity of colonic inflammatory bowel disease (IBD) is correlated with CCR9 and CCL25 [28]. A high-density lipoprotein diet (HFD) effectuated lesser IR in wild-type (WT) mice than their CCR9 gene knockout (KO) counterparts. Moreover, the expression of glucose metabolism-related genes was upregulated in CCR9KO mice [29]. Nonetheless, the relationship between CCL25/CCR9 and PCOS is yet to be investigated. FF constitutes a crucial microenvironment for oocyte development and maturation. Numerous inflammatory factors are active throughout the process, from primordial follicle development to preovulatory follicles [30]. In this study, we observed higher expressions of CCL25 and CCR9 in the FF of the HOR group compared to the NOR group. Subsequently, the correlation analyses showed that CCL25 was positively correlated with the number of oocytes retrieved and those with diameter ≥14 mm during COS, AMH, AFC, and HOMA-IR in the HOR group. In addition, CCR9 was positively correlated with the number of oocytes retrieved, and the number of oocytes with diameter ≥14 mm during COS, AMH, and AFC. Furthermore, multivariate linear regression analysis revealed that the elevated levels of AFC are independently associated with increased levels of CCL25. These findings indicate that CCL25/CCR9 may be involved in the inflammatory response in PCOS with HOR.

Several studies have shown that women with PCOS are in a state of low-grade chronic inflammation. The levels of cytokines (IL-6, IL-18, and TNF-*α*) were elevated in peripheral blood [31]. Additionally, IL-6, IL-8, and IL-18 concentrations in FF increased significantly and were positively correlated with fatty acid levels [12]. The augmentation of inflammatory mediators exhibited a correlation with obesity, IR, and androgen levels in PCOS patients, playing a role in the occurrence and progression of complications associated with PCOS [32]. Relevant studies implicated excessive inflammatory responses in OHSS, and IL-1, IL-6, and IL-8 levels were significantly elevated in the ascitic fluid [13]. Strikingly, the elevated level of serum IL-6 normalized gradually with improving symptoms. We also detected the expression levels of inflammatory cytokines IL-6, TNF-*α*, and IL-1*β* produced by CCR9-expressing immune cells in the FF of PCOS patients and observed the elevated levels of IL-6 and TNF-*α* in the HOR group, while no significant differences were detected in the IL-1*β* level between the two groups. These results substantiated that IL-6 and TNF-*α* were involved in the chronic low-grade inflammation in the pathomechanism of the HOR in PCOS.

The normal function of the ovaries is dependent on the periodic reconstruction of the extracellular matrix (ECM), and the cyclical changes in ECM play a crucial role throughout the processes of follicular development, ovulation, corpus luteum formation, maintenance, and regression. Additionally, ECM environmental stability largely depends on the coordinated control of MMPs and TIMPs [33]. MMP-2 and MMP-9 are the primary proteases that degrade ECM components and are the major regulatory factors in ovulation. Elevated MMP-2 and MMP-9 levels may indicate abnormal follicular development in PCOS patients. Previous studies showed higher concentrations of MMPs in the circulation and FF of PCOS patients compared to normal individuals [21, 34], which might be attributed to the abundant follicular reserves in PCOS patients; hence, a large amount of MMPs is required in ECM remodeling during IVF treatment. However, the relationship between MMPs and HOR has not yet been reported. Our study found increased levels of MMP-2 and MMP-9 in the FF in the HOR group; also, the levels of TIMP-2 and TIMP-1 were increased. Furthermore, the MMP-2 and MMP-9 expression levels were positively correlated with the number of oocytes retrieved, the number of oocytes with diameter ≥14 mm during COS, and AMH in the HOR group. A positive correlation was also established between MMP-9 and AFC. Based on these findings, we hypothesized that the MMP-TIMP system is involved in the pathological process of increased ovarian responsiveness in patients with PCOS treated with COS.

Notably, IR plays a crucial role in the pathogenesis of PCOS [35]. The evidence suggests that high HOMA-IR values are associated with an increased risk of developing OHSS among PCOS patients [36]. The current study observed a positive correlation between CCL25 levels in FF from PCOS patients and HOMA-IR values; moreover, HOMA-IR was identified as an independent influencing factor for increased CCL25 levels in FF from patients with HOR. Whether CCL25/CCR9 is associated with IR in PCOS is yet to be elucidated. Inflammatory cytokines and chemokines are associated with IR development through altered adipocyte insulin signaling molecules [37, 38]. Furthermore, the study investigating the role of the CCL25/CCR9 axis in the pathogenesis of type 2 diabetes identified a key role of CCR9 in inducing glycose intolerance via inducing the infiltration of CD4+ T cells in the small intestine [29]. In isolated human and mouse islets, exogenous CCL25 inhibited glucose-induced insulin secretion in a concentration-dependent manner and enhanced cytokine-induced apoptosis [14]. These results indicate that CCL25/CCR9 is associated with an increased risk of the HOR in PCOS with IR; however, the underlying mechanisms need to be clarified.

## 5. Conclusion

The current study shows that the levels of CCL25/CCR9, MMP-2, MMP-9, and related inflammatory factors are elevated in the FF samples obtained from PCOS patients with HOR undergoing COS. Correlation analysis reveals that CCL25, CCR9, MMP-2, and MMP-9 are associated with clinical indicators of HOR diagnosis. Multivariable linear regression analysis indicated that AFC and HOMA-IR are independent factors influencing the upregulation of CCL25. Together, these results suggest a significant role of CCL25/CCR9 in the pathogenesis of HOR in PCOS patients.

## Figures and Tables

**Table 1:** Baseline characteristics and COS of PCOS patients in different ovarian response groups (*n* = 200).

	HOR (*n* = 118)	NOR (*n* = 82)	*P* value
Age (y)	29 (26–32)	30 (28–33)	0.169
BMI (kg/m^2^)	25.08 ± 4.01	26.18 ± 4.35	0.069
AMH (ng/ml)	10.81 (6.85–14.38)	7.37 (3.97–10.14)	<0.001^∗^
AFC	24 (16–26)	15 (11–16)	<0.001^∗^
bFSH (mIU/mL)	6.45 (5.41–7.33)	7.05 (5.70–7.96)	0.03^∗^
bLH (mIU/mL)	6.64 (4.26–9.05)	5.25 (3.22–8.43)	0.217
bP (ng/ml)	0.59 (0.41–0.92)	0.51 (0.38–0.72)	0.09
bE2 (pg/mL)	38.5 (27.0–54.25)	37.00 (23.75–56.5)	0.725
bPRL (ng/ml)	13.70 (10.55–18.93)	13.50 (10.40–15.95)	0.358
bT (ng/dl)	58.13 (47.79–74.05)	53.99 (38.78–69.31)	0.051
HOMA-IR	4.00 (2.72–5.90)	3.21 (2.00–5.78)	0.039^∗^
Initial Gn dosage (IU/day)	225.0 (175.0–250.0)	225.0 (175.0–262.5)	0.632
Stimulation time (days)	10 (8–11)	10 (8–12)	0.716
Total dosage of Gn (IU)	1687.5 (1500–1912.5)	1800 (1675–1950)	0.111
Number of oocytes with diameter ≥14 mm during COS	20 (17–23)	11.5 (9–15)	<0.001^∗^
E_2_ on HCG day (pg/ml)	5743 (4767–8454)	2467 (1822–3483)	<0.001^∗^
Number of oocytes retrieved	16 (13–19)	10 (7.75–12)	<0.001^∗^

COS, controlled ovarian stimulation; PCOS, polycystic ovary syndrome; HOR, high ovarian response; NOR, normal ovarian response; BMI, body mass index; AMH, anti-Müllerian hormone; AFC, antral follicle count; bFSH, basal follicle-stimulating hormone; bLH, basal luteinizing hormone; bP, basal progesterone; bE_2_, basal estradiol; bPRL, basal prolactin; bT, basal testosterone; HOMA-IR, homeostasis model of assessment-insulin resistance; Gn, gonadotrophin. ^∗^*P* values <0.05.

**Table 2:** Correlations among study parameters in patients (values correspond to the *r* coefficient of correlation).

Patients	Number of oocytes retrieved	Number of oocytes with diameter ≥14 mm during COS	E_2_ on HCG day	AMH	AFC	bFSH	HOMA-IR
*HOR group*							
CCL25	**0.19 (** **p**=0.042**)**	**0.19 (** **p**=0.038**)**	0.07 (*p*=0.438)	**0.32 (** **p** < 0.001**)**	**0.33 (** **p** < 0.001**)**	−0.03 (*p*=0.760)	**0.40 (** **p** < 0.001**)**
CCR9	**0.21 (** **p**=0.020**)**	**0.27 (** **p**=0.003**)**	0.12 (*p*=0.178)	**0.26 (** **p**=0.004**)**	**0.35 (** **p** < 0.001**)**	0.00 (*p*=0.998)	0.02 (*p*=0.824)
MMP-2	**0.27 (** **p**=0.003**)**	**0.24 (** **p**=0.009**)**	0.16 (*p*=0.091)	**0.32 (** **p** < 0.001**)**	0.05 (*p*=0.579)	−0.06 (*p*=0.538)	0.07 (*p*=0.423)
MMP-9	**0.18 (** **p**=0.048**)**	**0.31 (** **p** < 0.001**)**	0.14 (*p*=0.119)	**0.44 (** **p** < 0.001**)**	**0.28 (** **p**=0.002**)**	−0.02 (*p*=0.847)	0.09 (*p*=0.337)

*NOR group*							
CCL25	**0.26 (** **p**=0.017**)**	0.10 (*p*=0.375)	0.16 (*p*=0.142)	**0.32 (** **p**=0.003**)**	0.19 (*p*=0.090)	−0.11 (*p*=0.342)	**0.29 (** **p**=0.008**)**
CCR9	**0.25 (** **p**=0.023**)**	**0.26 (** **p**=0.019**)**	**0.25 (** **p**=0.022**)**	**0.28 (** **p**=0.011**)**	0.06 (*p*=0.617)	−0.13 (*p*=0.244)	0.14 (*p*=0.212)
MMP-2	**0.24 (** **p**=0.032**)**	**0.26 (** **p**=0.016**)**	0.13 (*p*=0.252)	**0.23 (** **p**=0.039**)**	0.14 (*p* < 0.222)	0.04 (*p*=0.690)	0.05 (*p*=0.638)
MMP-9	**0.26 (** **p**=0.020**)**	**0.23 (** **p**=0.035**)**	0.06 (*p*=0.612)	0.14 (*p*=0.197)	0.06 (*p*=0.603)	0.04 (*p*=0.720)	0.04 (*p*=0.696)

Bold text indicates *P*  <  0.05; HOR, high ovarian response; NOR, normal ovarian response; COS, controlled ovarian stimulation; AMH, anti-Müllerian hormone; AFC, antral follicle count; HOMA-IR, homeostasis model of assessment-insulin resistance; MMP, metalloproteinase; TIMP, tissue inhibitor of metalloproteinase.

**Table 3:** Multivariable linear regression analysis of factors associated with elevated CCL25 levels in patients with HOR.

	Unstandardized coefficients		Standardized coefficients	*t*	*P*
	*B*	SE	Beta		
AMH	6.791	8.244	0.072	0.824	0.412
AFC	19.350	7.415	0.223	2.609	0.010^∗^
HOMA-IR	64.260	12.525	0.419	5.131	<0.001^∗^

SE, standard error; AMH, anti-Müllerian hormone; AFC, antral follicle count; HOMA-IR, homeostasis model of assessment-insulin resistance. ^∗^*p* values < 0.05.

## Data Availability

All data generated or analyzed during this study are included in this article.
